# Pre-analytical parameters associated with unsuccessful karyotyping in myeloid neoplasm: a study of 421 samples

**DOI:** 10.1590/1414-431X20188194

**Published:** 2019-02-14

**Authors:** M.F.M. Santos, F.C.A.C. Oliveira, R.K. Kishimoto, D. Borri, F.P.S. Santos, P.V. Campregher, P.A.A. Silveira, N. Hamerschlak, C.L.P. Mangueira, F.B. Duarte, A.H. Crepaldi, M.A. Salvino, E.D.R.P. Velloso

**Affiliations:** 1 Hospital Israelita Albert Einstein, São Paulo, SP, Brasil; 2 Hospital Universitário Walter Cantídio, Universidade Federal do Ceará, Fortaleza, CE, Brasil; 3 Hospital de Câncer de Mato Grosso, Cuiabá, MT, Brasil; 4 Hospital São Rafael/Monte Tabor, Salvador, BA, Brasil; 5 Hospital das Clínicas, Faculdade de Medicina, Universidade de São Paulo, São Paulo, SP, Brasil

**Keywords:** Cytogenetics, Karyotype, Leukemia, Myelodysplasia, Myeloproliferative disease

## Abstract

Cytogenetics is essential in myeloid neoplasms (MN) and pre-analytical variables are important for karyotyping. We assessed the relationship between pre-analytical variables (time from collection to sample processing, material type, sample cellularity, and diagnosis) and failures of karyotyping. Bone marrow (BM, n=352) and peripheral blood (PB, n=69) samples were analyzed from acute myeloid leukemia (n=113), myelodysplastic syndromes (n=73), myelodysplastic syndromes/myeloproliferative neoplasms (n=17), myeloproliferative neoplasms (n=137), and other with conclusive diagnosis (n=6), and reactive disorders/no conclusive diagnosis (n=75). The rate of unsuccessful karyotyping was 18.5% and was associated with the use of PB and a low number of nucleated cells (≤7×10^3^/µL) in the sample. High and low cellularity in BM and high and low cellularity in PB samples showed no metaphases in 3.9, 39.7, 41.9, and 84.6% of cases, respectively. Collecting a good BM sample is the key for the success of karyotyping in MN and avoids the use of expensive molecular techniques.

## Introduction

Cytogenetic study is essential for disease classification, prognostic assessment, and treatment (1). Typical chromosomal rearrangements are closely associated with specific tumor types, and the analysis of chromosomal abnormalities can be used to identify subpopulations that are most likely to benefit from specific treatments (specific drug targets) (2).

The World Health Organization recognizes genetic changes to define specific disease entities such as myeloid neoplasms (MN); myeloproliferative neoplasms (MPN); myelodysplastic/myeloproliferative neoplasms (MDS/MPN); myeloid and lymphoid neoplasms with *PDGFRα*, *PDGFRβ* or *FGRF1* or *PMC1-JAK2* abnormalities (MLN); myelodysplastic syndromes (MDS); and acute myeloid leukemias (AML) (3).

Karyotyping (obtained from conventional cytogenetics) remains an essential test for myeloid malignancies. It allows a comprehensive structural analysis of the complete set of chromosomes, though only anomalies comprising more than 5 MB can be detected. Chromosomes in metaphase are indispensable for karyotyping, and for this purpose, aspirated bone marrow or peripheral blood cells are cultured *in vitro*. The described rate of unsuccessful karyotyping in hematological malignancies samples is 10–20%. Some pre-analytical variables such as the time from collection to processing, the sample type (bone marrow or peripheral blood), the volume, the nucleated cell number, and other conditions intrinsic to the processing methods (culture, harvesting, and banding) must be met for successful analysis (4).

This study aimed to assess the relationship between pre-analytical variables and failures associated with karyotyping in myeloid neoplasms (MN).

## Material and Methods

### Case identification

This study was approved by the Ethics Committee of Hospital Israelita Albert Einstein (No. 08942912.0.1001.0071), and written informed consent was obtained from all of the participants. This was a national multicenter study with participation of twelve centers from five (Northeastern, Midwest, and Southeast) Brazilian states, and 358 patients with suspicion or diagnosis of MN were included from October 2012 to September 2014. Diagnosis was based on each patient’s clinical history, peripheral blood counts, hematopathology report, and results from flow cytometric immunophenotyping, and other molecular tests such as *BCR/ABL1* fusion, *PDGFRα* rearrangement, and *JAK2* mutation. WHO criteria were used for the final diagnosis (3).

### Cytogenetic methods

Bone marrow (BM, n=352) and peripheral blood (PB, N=69) samples were processed at the Cytogenetic Laboratory of Hospital Israelita Albert Einstein, São Paulo, Brazil. The transportation of samples from external centers was performed according to the standards and recommendations of the National Transportation Agency for biological materials.

Peripheral blood or bone marrow samples were cultured for 24 and 48 h without mitogenic agents and harvested following standard protocols (1).

### Pre-analytical variables

The following pre-analytical variables were studied: 1) time from collection to sample processing (more or less than 24 h); 2) material type (BM or PB); 3) sample cellularity (>7 or ≤7×10^3^/µL), and 4) patient's diagnosis (AML, MDS, MPN, or MDS/MPN).

### Statistical analysis

Chi-squared test or likelihood ratio test was used to verify associations of variables with karyotyping and not-adjusted OR with 95%CI was estimated using bivariate logistic regression. Multivariate logistic regression was performed to adjust all variables in the model. The data were analyzed using IBM SPSS Statistics software (USA). The level of significance for the statistical tests was 5% (P value <0.05).

## Results

Three hundred and fifty-eight patients with a median age of 60 (16-86) years (52% female) were analyzed; some patients had serial samples. From the 421 samples sent to the laboratory, 340 (80.8%) samples belonged to patients with acute or chronic myeloid neoplasms with the following confirmed diagnoses: 1) 137 (32.5%) MPN; 2) 113 (26.8%) AML; 3) 73 (17.3%) MDS; and 4) 17 (4.0%) MDS/MPN. Most of the samples from the MPN group belonged to patients with myelofibrosis (MF, n=58), essential thrombocythemia (n=41), polycythemia vera (n=17), and non-categorized MPN (n=21). The other samples belonged to patients with MLN with a *PDGFRα* rearrangement (1 case), lymphoid neoplasms (5 cases: 1 ALL, 1 CLL, 1 hairy cell leukemia, and 2 NHL), or reactive disorders/non-conclusive final diagnoses (75 cases of thrombosis, polycythemia, eosinophilia, anemia).

Two hundred and eighty-two (67.0%) samples were collected at the Hospital Israelita Albert Einstein, while 139 (33.0%) samples came from 12 different centers. The median time from sample collection to culture processing was 24 h (306 samples in 24 h, 67 in 24–48 h, 33 in 48–72 h, 15 in >72 h). In 94 cases, there were less than 7×10^3^ nucleated cells per microliter of sample: 68 in BM and 26 in PB.

There were no evaluable metaphases (unsuccessful karyotyping – UK) in 78 (18.5%) samples. The median number of metaphases analyzed was 20 (from 10 to 30) and only 23 cases presented less than 20 metaphases. There was no significant difference from samples processed in less than 24 h (52/306, 17% of no metaphases), from 24 to 48 h (14/67, 20.9%), and more than 48 h (12/48, 25%). The following pre-analytical variables were associated with UK: 1) peripheral blood as a sample (P<0.001); 2) low cellularity (≤7×10^3^/μL) samples (P<0.001), and 3) diagnosis (P=0.018) (Supplementary Table S1). High and low cellularity in BM and high and low cellularity in PB samples showed no metaphases in 3.9, 39.7, 41.9, and 84.6% of cases, respectively. The diagnosis showed no significant differences based on the success of karyotyping when the “others” category was removed from the analysis. Most of the UK was recognized as related to low cellularity (49 samples – 62.8%) or peripheral blood (40 samples – 51.3%), although insufficient volumes (5 samples – 6.4%) and aged samples (3 samples – 3.8%) were also detected. Technical problems resulting in poor quality of metaphases were detected in 3 (3.8%) cases. The multivariate analysis confirmed that only cellularity and type of sample were relevant (P<0.001) for successful karyotyping in myeloid neoplasms.

## Discussion

Cytogenetic studies are important for accurate diagnosis, appropriate treatment, and monitoring the response to therapy. Many of these aberrations have emerged as prognostic and predictive markers in hematologic cancers. Despite its importance, sometimes G-banding karyotyping cannot be performed due to technical difficulties such as low mitotic index and sample type collected.

The rate of unsuccessful karyotyping in this study was 18.5%, similar to the described rate of UK in hematological malignancy (10–20%) (4). In this study comprising only MN, the UK was associated basically to the low number of nucleated cells and the PB samples.

Concentration of 1 million cells/1 mL of medium is optimal, and most laboratories, including ours, attempt to obtain 1–2 ×10^3^ cells/µL (10^6^ cells/mL) culture, suspending the sample in 5–10 mL growth medium (5). Low cellularity was significantly associated with higher frequency of UK. If cell counts are low, guidelines (5,6) suggest a culture of lower volume in order to maintain the cellular concentration. At least two different cultures are recommended, using two different media or two different culture times.

Although small or poor quality samples can sometimes fail to provide enough divisions, the high-count samples are most likely to fail completely. The vast majority of these cells are incapable of division, and their presence inhibits the few remaining cells that can divide (7). High cellularity BM sample showed UK in only 3.9%, but in PB this rate was 41.9%.

Non-evaluable metaphases in bone marrow samples were 10.8% (38/352) of cases. Some cases with UKs are undoubtedly due to insufficient cell number in the bone marrow aspirates, which is often the case in samples from AML with myelofibrosis and hypocellular AML. This result could possibly be remedied by using bone marrow biopsies (8). Despite this, in some circumstances, blood culture should be considered an alternative where a BM sample or culture has proved inadequate. Blood culture is not appropriate for all diagnoses, like MDS, MPD (except chronic granulocytic leukemia and myelofibrosis), or pancytopenic AML (9). For instance, aspiration of BM is often unsuccessful in MF, because of considerable fibrotic changes and replacement of hemopoietic cell clusters onto reticulin and collagen fibers (10). Peripheral blood specimens may yield informative results when the circulating blast cell percentage is higher than 10%. In general, the abnormal clone can be identified in such specimens, albeit not as often as in bone marrow (6). On the other hand, bone marrow samples that have been contaminated with blood might not have an adequate number of spontaneously dividing cells. For this reason, it is important that the cytogenetics laboratory receive the first few milliliters of the bone marrow tap (11).

Peripheral blood samples were significantly associated with higher frequency of UK. Hussein et al. (12) reported successful karyotyping in 42% (102/242) of the cases of PB cytogenetics. In that study, differently from our findings, white blood cells did not independently predict the success of obtaining peripheral blood metaphases. Success was associated with an increasing level of myeloid progenitor cells or blasts of either myeloid or lymphoid lineage (P=0.0007) and with abnormal BM cytogenetics (P=0.005) (12). A study of BM and PB samples from patients with MF with cytogenetic analysis of PB samples without stimulation of cell division was unsuccessful in all 10 patients due to either insufficient quantity or quality of metaphase plates, or lack of mitosis in the samples (10). However, Lozynskyy et al. (10) set up cell cultures of PB leukocytes stimulated *in vitro* with G-CSF and all 31 patients resulted in successful karyotyping. Moreover, chromosome abnormalities were detected in 19 (45.2%) of the patients in cell cultures of PB leukocytes stimulated *in vitro* with G-CSF, and in non-stimulated BM samples, abnormalities were detected in 19 (45.2%) of all the patients, demonstrating that PB studies stimulated with appropriate mitogens may reduce the need of painful invasive diagnostic manipulations and facilitate follow-up of the patients.

Specimens should be received by the laboratory as soon as possible, without exposure to extreme temperatures, ideally within 24 h, and the use of transport medium is strongly recommended to minimize drying-out of the sample and to maintain the viability of the cells (6,9). There was no statistical significance regarding the time from collection to sample processing, all samples were sent to the laboratory in transport culture medium, and 88.6% set up cell cultures within 48 h. In addition to time, other factors can influence the culture, such as sample type and cell concentration. A result can sometimes be obtained even from samples a few days old, with myeloid disorders being generally more tolerant of delay than samples from lymphoid disorders, and samples with a high white blood cell count usually need prompt attention (7). In this study, only a few “aged samples” were received; however, three of the UK cases were detected as “aged samples”. Besides human errors in taking the bone marrow aspirates, such as volumes that are insufficient for performing at least two cellular cultures and diluting the bone marrow cells with peripheral blood, technical problems in the laboratory must be taken into account, such as equipment failure or preparation of reagents.

Diagnosis had no statistical impact on the success of karyotyping with the removal of the samples from the “other” category, so the diagnosis was not used in the final model. Our incidence of UK was 13.3 and 16.4% in AML and MDS, respectively. Incidence of an UK in AML is 10% in the literature and is related to poor prognosis (P=0.002) (13). In MDS, UK is 6–7%, mainly in patients with fibrotic or hypocellular marrows (14). In AML, UK occurs more commonly in older patients, predicts poor response to chemotherapy, and should be considered a high-risk feature (13). Study of FISH with MDS cases with G-banding failure did not identify abnormalities with poor prognosis and none of the patients had features of high risk MDS by morphologic criteria suggesting that this finding is associated with indolent forms of MDS (15). In contrast, in Cervera's study (16), unsuccessful conventional cytogenetic analysis in MDS was associated with worse survival compared to normal karyotyping.

Unsuccessful conventional cytogenetic analysis is directly related to cellularity and type of sample, so collecting a good and adequate sample is key for karyotyping success in myeloid neoplasm.

## Supplementary Material

Click here to view [pdf].
